# PD_NGSAtlas: a reference database combining next-generation sequencing epigenomic and transcriptomic data for psychiatric disorders

**DOI:** 10.1186/s12920-014-0071-z

**Published:** 2014-12-31

**Authors:** Zheng Zhao, Yongsheng Li, Hong Chen, Jianping Lu, Peter M Thompson, Juan Chen, Zishan Wang, Juan Xu, Chun Xu, Xia Li

**Affiliations:** College of Bioinformatics Science and Technology, Harbin Medical University, Harbin, 150081 China; Department of Pediatrics, Paul L. Foster School of Medicine, Texas Tech University Health Science Center, El Paso, TX USA; Southwest Brain Bank, Department of Psychiatry, UTHSCSA, San Antonio, TX USA

**Keywords:** Schizophrenia, Bipolar disorder, Next-generation sequencing, Epigenomic and transcriptomic data, Brain, Blood

## Abstract

**Background:**

Psychiatric disorders such as schizophrenia (SZ) and bipolar disorder (BP) are projected to lead the global disease burden within the next decade. Several lines of evidence suggest that epigenetic- or genetic-mediated dysfunction is frequently present in these disorders. To date, the inheritance patterns have been complicated by the problem of integrating epigenomic and transcriptomic factors that have yet to be elucidated. Therefore, there is a need to build a comprehensive database for storing epigenomic and transcriptomic data relating to psychiatric disorders.

**Description:**

We have developed the PD_NGSAtlas, which focuses on the efficient storage of epigenomic and transcriptomic data based on next-generation sequencing and on the quantitative analyses of epigenetic and transcriptional alterations involved in psychiatric disorders. The current release of the PD_NGSAtlas contains 43 DNA methylation profiles and 37 transcription profiles detected by MeDIP-Seq and RNA-Seq, respectively, in two distinct brain regions and peripheral blood of SZ, BP and non-psychiatric controls. In addition to these data that were generated in-house, we have included, and will continue to include, published DNA methylation and gene expression data from other research groups, with a focus on psychiatric disorders. A flexible query engine has been developed for the acquisition of methylation profiles and transcription profiles for special genes or genomic regions of interest of the selected samples. Furthermore, the PD_NGSAtlas offers online tools for identifying aberrantly methylated and expressed events involved in psychiatric disorders. A genome browser has been developed to provide integrative and detailed views of multidimensional data in a given genomic context, which can help researchers understand molecular mechanisms from epigenetic and transcriptional perspectives. Moreover, users can download the methylation and transcription data for further analyses.

**Conclusions:**

The PD_NGSAtlas aims to provide storage of epigenomic and transcriptomic data as well as quantitative analyses of epigenetic and transcriptional alterations involved in psychiatric disorders. The PD_NGSAtlas will be a valuable data resource and will enable researchers to investigate the pathophysiology and aetiology of disease in detail. The database is available at http://bioinfo.hrbmu.edu.cn/pd_ngsatlas/.

**Electronic supplementary material:**

The online version of this article (doi:10.1186/s12920-014-0071-z) contains supplementary material, which is available to authorized users.

## Background

Schizophrenia (SZ) and bipolar disorder (BP) are common and highly heritable psychiatric disorders that affect approximately 4% of the world’s population and result in considerable personal and societal burdens [1]. Over the past decades, it has been widely accepted that both genetic and environmental risk factors lead to the occurrence and development of these disorders [2-4]. Moreover, a large number of genetic association and linkage studies have been performed to explore the pathogenesis of SZ and BP [5,6]. However, the results do not replicate well, and they identify risk alleles with small effects, indicating that non-genetic factors may also result in disease [7]. Recent studies have highlighted a role for epigenetic processes in mediating susceptibility, and have provided new insight into disease pathogenesis.

DNA methylation, which consists of the addition of a methyl group to the 5’-position of cytosine in CpG dinucleotides, is an important epigenetic modification involved in the regulation of transcription [8]. DNA methylation has been shown to interfere with transcription by directly inhibiting the binding of transcription factors, enhancering blocking elements, or recruiting methyl-CpG binding proteins (MBPs) to affect chromatin structure [9]. DNA methylation plays a crucial role in genomic imprinting, X chromosome inactivation and regulating tissue-specific gene expression [8,10,11]. Accumulating evidence indicates that abnormal DNA methylation at particular locations may affect neuronal activity [12], brain growth and development [13], learning and memory [14], and cognitive performance [15], and is associated with the pathophysiology of psychiatric disorders [16]. Initial studies focused on DNA methylation alterations in some candidate genes. Using cultured rat neurons, Chen *et al.* and Martinowich *et al.* showed the importance of DNA methylation in the regulation of brain-derived neurotrophic factor (BDNF), which is essential for neuronal survival, development and synaptic plasticity [17,18]. Subsequently, the first genome-wide DNA methylation landscape profiled by Mill *et al.* aimed to investigate DNA methylation changes associated with SZ and BP using CpG-island microarrays of approximately 12,000 GC-rich regions in the prefrontal cortex and in the germline [19]. They found evidence for psychosis-associated DNA methylation differences in numerous loci involved in glutamatergic and GABAergic neurotransmission, brain development, and other processes functionally linked to disease aetiology. Because monozygotic (MZ) twins share common genetic information and can be used as an ideal model for investigating the contribution of epigenetic factors to disease aetiology, Dempster *et al.* performed a genome-wide analysis of methylation of DNA in blood samples from MZ twin pairs discordant for major psychoses using microarrays, and they demonstrated disease-associated DNA methylation differences between twins.

Although epigenetic studies promote our understanding of psychiatric disorders, there have been few studies of methylation and gene expression on a genome-wide scale. Initial studies focused on DNA methylation alterations in candidate genes, including RELN [20], SOX10 [21] and GAD67. DNA methylation of the reelin promoter was suggested to be involved in downregulating the gene in SZ, and the DNA methylation status of SOX10 inversely correlated with expression levels of SOX10 and other oligodendrocyte genes [21]. Rapid advances in the development of next-generation sequencing (NGS) technology facilitates correspondingly dramatic advances in elucidating how epigenetic processes mediate gene expression and makes it possible to integrate epigenomic and transcriptomic data to uncover the aetiology and pathophysiology of psychiatric disorders. Recently, we performed genome-wide methylation and expression analyses in two brain regions and in peripheral blood samples [22,23]. Our results support the important roles of DNA methylation in SZ and BP and highlight the complex relationships between DNA methylation and gene expression in these disorders. In addition, the results indicate that differentially expressed genes with aberrant methylation patterns that we identified may represent novel candidates for the aetiology and pathology of neuropsychiatric disorders.

To our knowledge, although a handful of DNA methylation databases have been compiled, they either contain limited methylation data or differ in biological scope. Among these methylation databases, NGSmethDB [24] and MethBank [25] were constructed to store genome-wide methylomes. However, MethBank only supports the storage, browsing and visualizing of whole-genome DNA methylation data in two well-studied species, D.rerio and M. musculus. In addition, NGSmethDB provides data sets for cell lines, fresh and pathological tissues but not for specific diseases. Several methylation databases centred on human diseases have also been compiled, including DiseaseMeth [26] and the Cancer methylome system (CMS) [27]. DiseaseMeth is a web-based resource focused on the aberrant methylomes of human diseases. However, most of the datasets are microarray-based. CMS is a web-based database application that provides comprehensive and genome-wide epigenetic portraits of human breast cancer and endometrial cancer. However, there is limited, specialised and comprehensive database of psychiatric disorders that focuses on the storage of epigenomic data based on next-generation sequencing. MethylomeDB [28] is the only database that presents methylation profiles of carefully selected non-psychiatric control, schizophrenia, and depression samples. However, the gene expression levels in these sample have not been profiled, and the database has not been updated for a long time. Thus, a reference database combining epigenomic and transcriptomic datasets is urgently needed for the combined analyses of the potential pathogenesis mechanisms of psychiatric disorders.

In this study, we developed the PD_NGSAtlas, which aims to store next-generation sequencing epigenomic and transcriptomic data captured from the same individuals and to perform quantitative analyses of epigenetic and transcriptional alterations involved in psychiatric disorders. The current version of the PD_NGSAtlas provides internal genome-wide DNA methylation and transcription profiles from two generally inaccessible brain regions and from accessible peripheral blood of SZ, BP and non-psychiatric disorder controls. The PD_NGSAtlas supports the search of methylation and transcription profiles for special genes or genomic regions of selected samples, which should enable a broad range of researchers to explore the molecular mechanisms of psychiatric disorders (Additional file 1: Figure S1). All retrieved results can be downloaded freely for further analysis. Furthermore, the PD_NGSAtlas offers online tools for identifying aberrantly methylated and expressed genes involved in psychiatric disorders. The database also features a genome browser, which can be used to browse multidimensional data in a given genomic context. In summary, the PD_NGSAtlas is a user-friendly, web-based, ‘one-stop’ service for basic data retrieval, analyses, visualisation and downloading, which will help provide new insights into the aetiology of psychiatric disorders.

## Construction and content

### Clinical samples

All of the subjects were diagnosed by consensus for either BP or SZ according to DSM-IV-TR criteria and the control samples had no history of an Axis I disorder. The diverse types of clinical characteristics were also collected, including disease status, disease types, age, age of onset, sex and twin status (Additional file 2: Table S1). All the subjects in this study were free of confounding neuropathology. DNA and RNA samples were obtained from peripheral blood or from two distinct brain regions. DNA and RNA samples of peripheral blood were obtained from the Department of Psychiatry and Center of Excellence – Neurosciences, Texas Tech University Health Science Center (TTUHSC), whereas the post-mortem brain tissues were collected from the Southwest Brain Bank (SWBB), Department of Psychiatry, UTHSCSA, TX USA. Written, informed consent was obtained from all the participants. All of the brain samples were from freshly frozen specimens that were stored in −80°C freezers. Brodmann area 9 (BA9) and BA24 from the same hemisphere were both used based on the criteria described by Rajkowska and Goldman-Rakic [29].

For all the samples stored in the PD_NGSAtlas, a tooltip was added that appears when hovering over a potential sample selection and lists its full parameters. Moreover, users can click on the sample item in the ‘Tools’ section to see its detailed clinical information that helps to better explore the nature of disease.

### MeDIP-Seq

The current release of the PD_NGSAtlas contains 43 DNA methylation profiles detected using MeDIP-Seq by our laboratory. The extracted genomic DNA samples were fragmented into 100-500bp by sonication. DNA ends were repaired to overhang a 3’-dA, and adapters were ligated to the DNA fragment ends. The double-stranded DNA was denatured, and the DNA fragments were immunoprecipitated using a 5-mC antibody. Real-time PCR was used to validate the immunoprecipitation quality. DNA fragments of the proper size (usually 200–300 bp, including the adapter sequence) were selected after PCR amplification. Finally, the resultant libraries were sequenced as paired-end 50 bp reads using the genome-wide massively parallel sequencing platform Illumina HiSeq 2000.

### RNA-Seq

RNA-Seq was performed to profile gene expression in 37 samples, including 14 SZ, 12 BP and 11 control samples. Oligo (dT) beads were used to isolate poly(A) mRNA from the total RNA from these samples. Fragmentation buffer was added and the resulting 200–300 bp fragments were used as templates for random hexamer-primer synthesis of first-strand cDNAs. Second-strand cDNA was synthesised using buffer, dNTPs, RNase H and DNA polymerase I. Fragments were purified using a QIAquick PCR extraction kit and eluted with EB buffer for end reparation and poly(A) addition. Based on the results of agarose gel electrophoresis, fragments were connected with sequencing adapters; PCR was performed by selecting suitable fragments as templates. The library was sequenced as paired-end 90 bp reads using an Illumina Hiseq 2000.

### Genomic features annotation

The genomic coordinates for the human genomic features investigated were downloaded from the UCSC table browser [30]. RefSeq gene promoters were defined as ±2 kb of sequence flanking the transcription start sites. Table CpGislandext (UCSC) was used for the set of CpG islands (CGIs). We excluded CGIs with ‘random’ chromosome locations. Following Andrew *et al.*, the CpG island shores were defined as the 2 kb regions near the CGIs. In addition, some histone modifications and open chromatin datasets were obtained from the ENCODE project [31] (Table 1). All the coordinates of the epigenomic and transcriptomic datasets and genomic features have been remapped from NCBI36/hg18 to GRCh37/hg19 using the UCSC’s liftOver tool.

**Table 1:** **All data content and statistics used in PD_NGSAtlas**

**Data content**	**Data statistics**	**Data description**	**Data sources**
DNA Methylation data	50	Include distinct brain regions and peripheral blood of SZ, BP and controls	MeDIP-Seq data
Transcriptomic data	146	Include distinct brain regions and peripheral blood of SZ, BP and controls	RNA-Seq data
Genome information (Hg19)	30	Include reference genome and the 29 genomic functional elements from UCSC table browser	UCSC table browser
Reference genome	1	DNA sequence	UCSC table browser
Genomic functional elements	29	Include reference genome, gene-, RNA-, CpG island- and repeat-related functional elements	UCSC table browser
Gene-associated	9	Include CDS, downstream2k, exon, five-UTR, intron, romoters, refseq gene and three-UTR, upstream2k	UCSC table browser
RNA-related	9	Include RNA, lincRNA, miRNA, miRNA promoter, rRNA, scRNA, snRNA, srpRNA and tRNA	UCSC table browser
CpG island-related	3	Include CpG islands, five-shores and three-shores	UCSC table browser
Repeat-related	8	Include LINE, LTR, low complexity, SINE, satellite, simple repeat, unkown repeat and DNA repeat	UCSC table browser
Regulation data	12	The 12 regulation data obtain from ENCODE project, including DNA methylation, histone and open chromatin data	ENCODE project
DNA methylation-related	3	DNA methylation regulation data involved in GM12878 H1-hESC and K562 cell lines using Methyl-seq	ENCODE project
Histone-related	6	Include H3K4Me1 and H3K4Me3 involved in GM12878 H1-hESC and K562 cell lines	ENCODE project
Open chromatin-related	3	Open chromatin data involved in GM12878 H1-hESC and K562 cell lines using DNase-seq	ENCODE project

### Genome-wide DNA methylation and transcription profiles

From the raw fastq files, Illumina quality scores were converted into Sanger Phred quality scores using MAQ. Quality control was performed on the raw sequence data using FastQC. Additional file 3: Figure S2 highlights the quality of our sequencing datasets. Reads from MeDIP-Seq and RNA-Seq were mapped using the SOAP2 program [32]. The uniquely mapped reads were retained for further analysis. The genome methylation peaks were further identified by MACS [33], and the threshold of the p-value was set to 1.0e-5. In addition, gene expression levels were measured using RPKM [34]. Finally, all the DNA methylation profiles cover 6,634,043 methylation peaks, and the transcription profiles involve 19,186 expressed genes.

**Figure 1:**
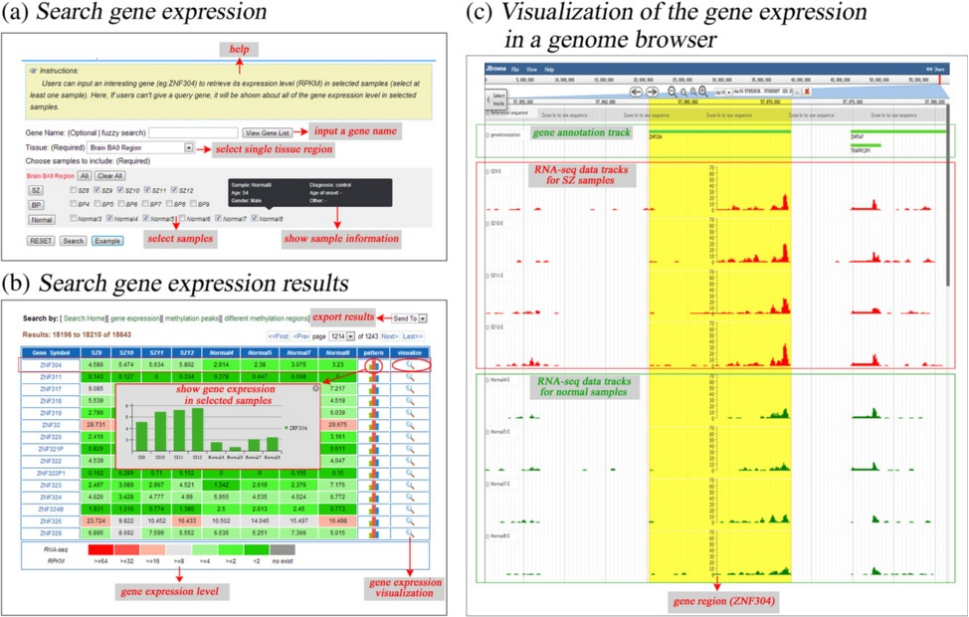
**The view of gene expression across samples. (a)** The search page allows the user to search the specific gene expression profile across samples. A tooltip shows the sample information. **(b)** The search result page displays the heat map of gene expression levels across the samples. A detail information will show the gene expression in selected samples when the user click the bar plot. **(c)** Visualization of the gene expression in a genome browser.

**Figure 2:**
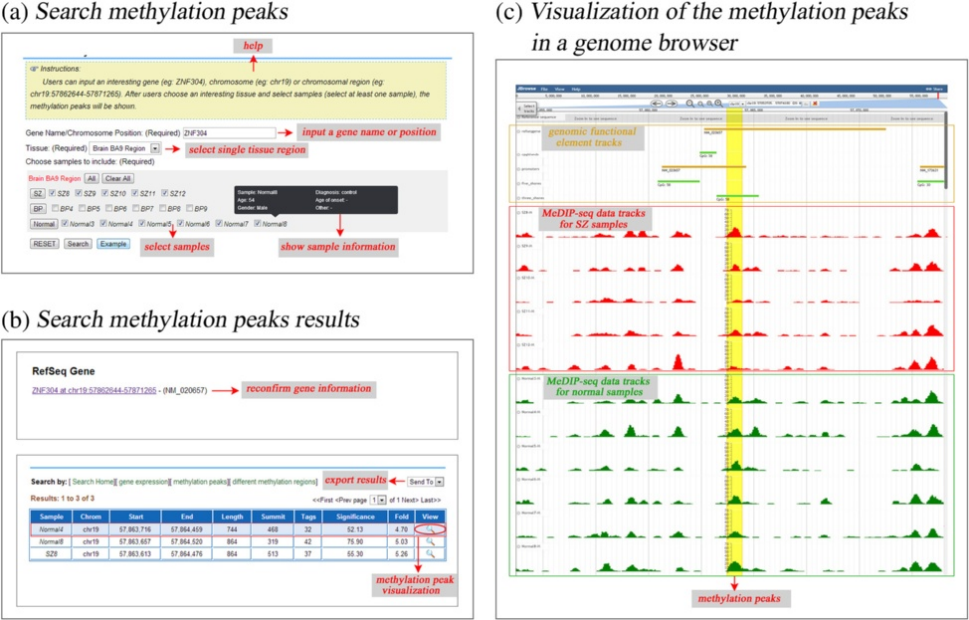
**The view of DNA methylation peaks across samples. (a)** The Search page allows the user to search the DNA methylation of specific gene across samples. A tooltip shows the sample information. **(b)** The search result page displays the DNA methylation peaks across the samples. **(c)** Visualization of the DNA methylation in a genome browser.

The PD_NGSAtlas provides a user-friendly interface for the acquisition of methylation profiles and transcription profiles for specific genes or genomic regions of selected samples. A comprehensive search interface is provided (Figure 1a). For transcription data, users can search gene expression levels by entering a gene symbol (optional) and selecting several samples of interest (Figure 1a). The search results are displayed as an overview table that summarises the gene expression levels across selected samples (Figure 1b). This table can show the gene expression pattern across selected samples and can link to the ‘Visualize’ section in which users can view gene expression profiles under a given genomic context through a tailored genome browser (Figure 1c). Similarly, users can obtain DNA methylation profiles of a given gene symbol or chromosome region across selected samples (Figure 2). Furthermore, these DNA methylation profiles can be visualised through a customised genome browser. All of the above query results can be downloaded freely. These valuable data resources should facilitate researcher on psychiatric disorders.

### Identification of aberrantly methylated and/or expressed events in psychiatric disorders

**Figure 3:**
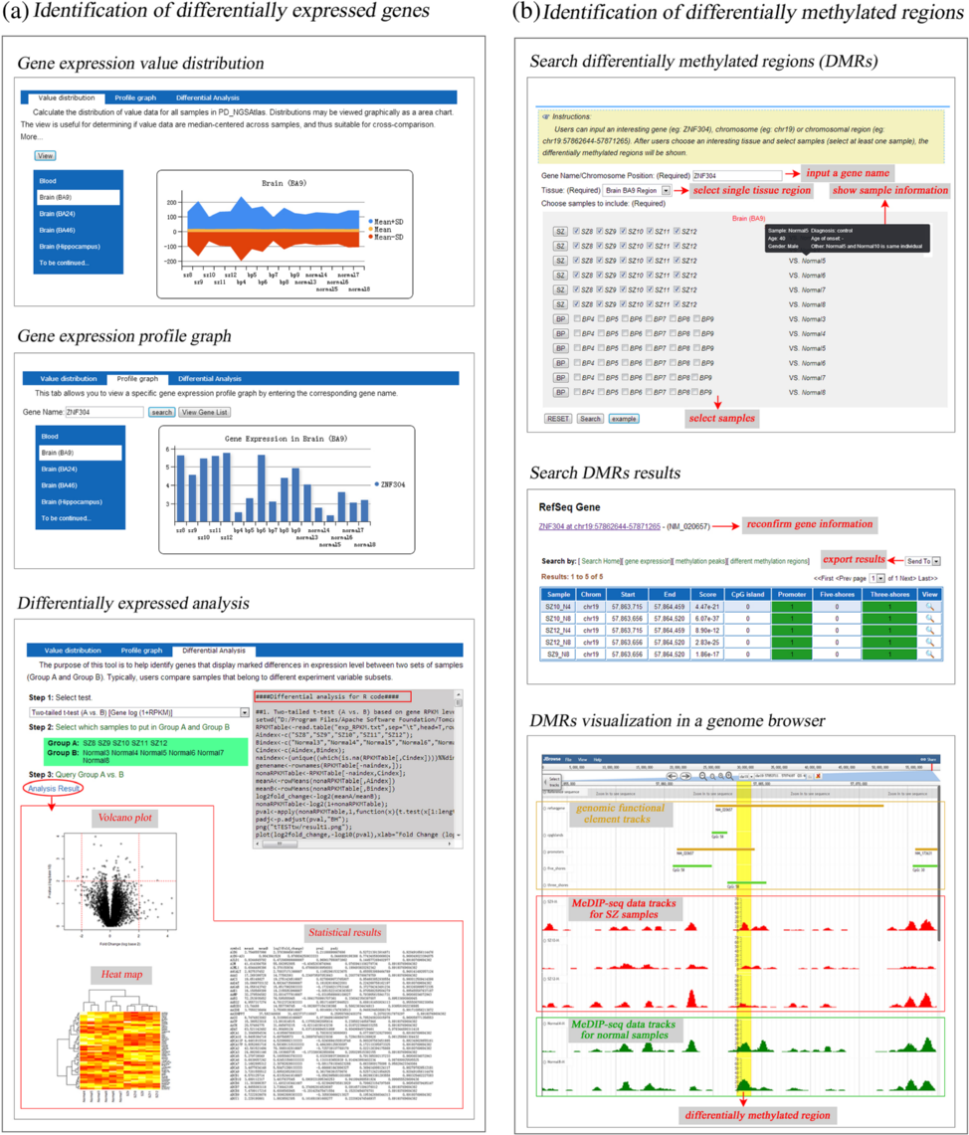
**The view of DEGs and DMRs across samples. (a)** The overview of the identification of DEGs. The upper panel shows the distribution of gene expression value data for all samples in PD_NGSAtlas. The middle panel allows the user to view a specific gene expression profile graph across samples by entering the corresponding gene name. The lower panel shows the tools incorporated in the database to identify the DEGs. **(b)** The overview of the identification of DMRs. The upper panel shows the search page of DMRs and the search results were shown in the middle panel. The users can view the DMR in a genome browser by clicking the view button.

In the PD_NGSAtlas database, to view global gene expression profiles, online tools can calculate the overall distribution of gene expression and present it graphically as a flex area chart (Figure 3a). The tool is useful for determining whether data values are median-centred across samples and thus suitable for cross-comparison. Similarly, users can type in a specific gene symbol and view its expression distribution across all samples in which its expression changes (Figure 3a). Typically, users can compare samples that belong to different experimental variable subsets. For transcription data, a tool was developed for users to identify genes that display marked differences in the expression levels of two sets of samples. In the current version of the database, a two-tailed t-test and several other widely used methods (including EdgeR [35] and DEGseq [36]) were provided to identify the differentially expressed genes (DEGs). The t-test is the most commonly used method to identify DEGs. With the development of high-throughput sequencing, several R packages were developed to identify DEGs for RNA-seq data. EdgeR integrated three existing methods and introduced two novel methods based on MA-plots to detect and visualise gene expression difference, whereas DEGseq used empirical Bayes methods to moderate the degree of overdispersion across transcripts, improving the reliability of inference. In addition, the Limma method can be used to identify the DEGs accounting for age and sex. All the p-values obtained by these methods were adjusted. In addition, the results of the DEGs are shown in a volcano plot, an M-A plot and a heatmap is provided to show the expression of the top 50 DEGs (Figure 3a). For DNA methylation data, aberrantly methylated peaks were detected between two samples. For each peak, the number of reads for each sample was calculated, and the significance was assessed using chi-squared tests. Then, the resultant regions with an FDR less than 5% and more than a two-fold difference of read numbers were considered to be differentially methylated regions (DMRs) [37]. In the PD_NGSAtlas, a query interface was designed to enable a comparison between disease samples and controls, which users can employ to obtain DMRs (Figure 3b). We propose that the combination of aberrantly methylated regions and expressed genes can be used to elucidate the molecular mechanisms underlying psychiatric disorders.

### Visualizing the methylation and transcription profiles of interesting genes and regions

**Figure 4:**
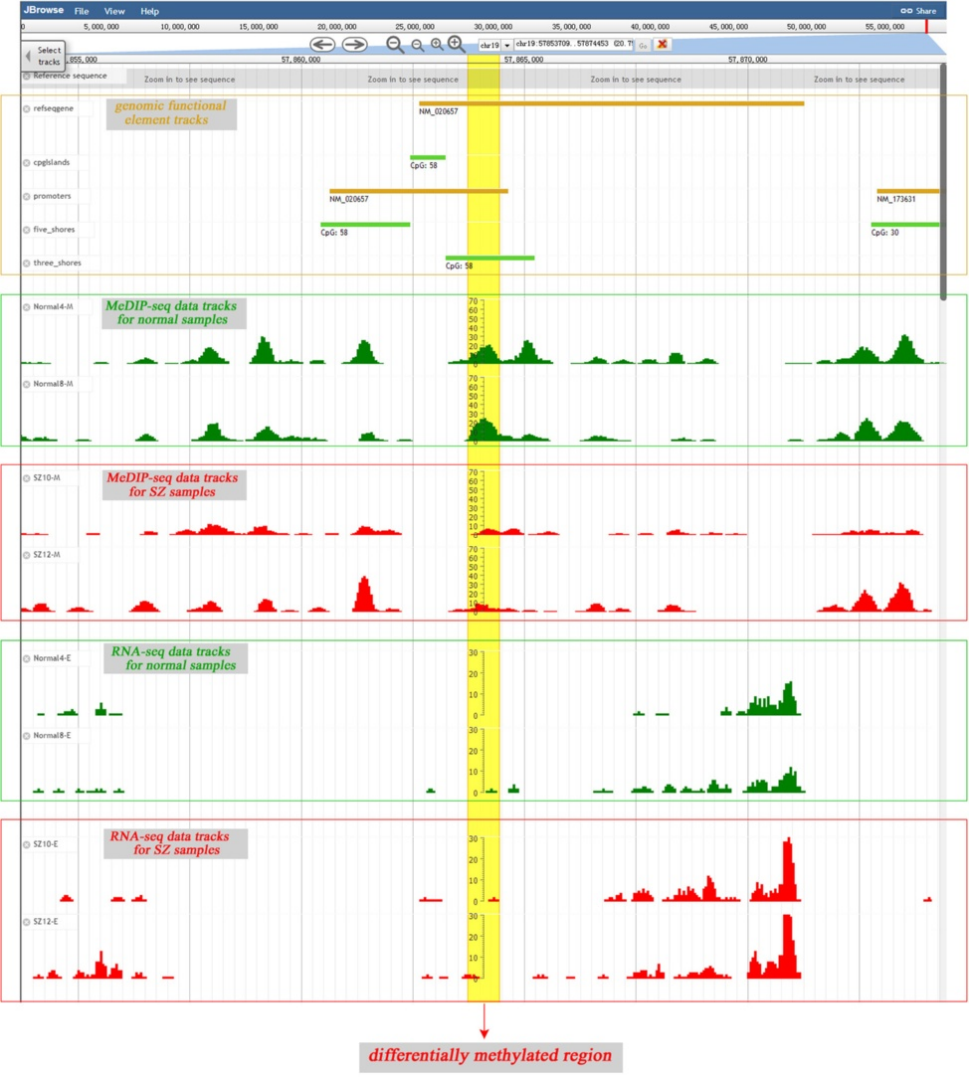
**Gene centric view of DMR.** The promoter of ZNF304 is hypomethylated in SZ samples compared with controls. The DMR was colored in yellow in the figure.

To capture meaningful information from epigenetic and transcriptomic data, a genome browser based on JBrowse was proposed for users, and it allowed users to compare multilevel genomic, epigenetic and transcriptomic data visually to discover functional relationships (Figure 4) [38]. Here, both methylation and transcription data can be visualised in the same view in bigWig format, which can help users to find the functional relationships between the two types of data. Furthermore, users can view other genome information including gene structure, CpG islands, repeat elements and several genomic regulation features against a human reference genome (Hg19). These data can intuitively reflect epigenetic and transcriptomic changes between different samples, which would be useful for the study of the molecular mechanisms of psychiatric disorders. The genome browser offers several easy-to-use tools, including the ability to navigate directly to a region of interest by typing in the region coordinates, to zoom in or out or drag a region, to view the annotation details by double-clicking on the annotation track, and to configure genomic annotation by clicking on the track name. Importantly, users can upload their own data to be visualised. The users’ data reside on a local computer without the need to transfer any data to the server. As shown in Figures 1–2, a visual interface can be accessed through the links in the query results.

### Relational database and web interface

The web interface was developed in Java using the Servlet framework. The PD_NGSAtlas website is deployed on a Tomcat 6.0.33 web server and runs under the Cent OS 5.5 system. It is supported by a MySQL database of DNA methylation and transcription data. The JQuery was used to render, generate and manipulate the gene expression distribution views. The module for the identification of differentially expressed genes (DEGs) is realised by R and Perl script. In the ‘Visualize’ module, JBrowse (release 1.11.5), an open source genome browser, can be used to navigate multiple omics data and diverse genome information over the web. Moreover, the PD_NGSAtlas has been fully tested in Google Chrome (version 17 and later), Apple Safari (version 5 and later) and Mozilla Firefox (version 10 and later).

## Utility

It is worth noting that the integration of epigenetic and transcriptomic data is intended to enhance the analysis of the aetiology of psychiatric disorders at the gene level. Taking the gene ZNF304 as an example, in the BA9 region, ZNF304 is specifically upregulated in patients with SZ compared with controls (Figure 4, t-test, p<10e-3). Furthermore, we found that the promoter of ZNF304 is hypomethylated in SZ samples compared with controls (Figure 4). In addition to ZNF304, we found that the expression of gene ZNF483 is higher in SZ samples than in the controls, and the promoter of ZNF483 is hypomethylated in SZ samples from the BA24 region of the brain. This is consistent with previous research implicating ZNF483 in SZ [39,40]. These results suggest that the combination of epigenetics and transcriptome studies may provide new insights into the cause of psychiatric disorders.

## Discussion

The current version of the PD_NGSAtlas is the first release of our database, and it contains next-generation sequencing DNA methylation and gene expression profiles of datasets obtained from human brain and blood samples. Psychiatric disorders are diseases of the central nervous system, and therefore, studies of patient-derived living brain cells may provide the most pertinent information. Post-mortem brains have been extensively used in recent studies; however, obtaining a sufficient number of brains in ideal condition is difficult. Thus, it is more feasible to obtain peripheral samples that can act as potential biomarkers of SZ and BP [41]. Psychiatric disorders, including SZ and BP, have genetic components [42], and CNS alterations might be reflected in peripheral tissues. Indeed, previous microarray analyses have found numerous classes of genes that are expressed both in blood and in the prefrontal cortex [43], including approximately half of the so-called SZ susceptibility genes [44]. A previous study comparing the methylation status of pre-mortem blood and post-mortem brain tissue showed that significant variation in the methylation profiles of brain tissue were reflected in blood [45]. Additionally, recent studies have shown that DMRs associated with both chronic pain and ageing are similar in brain and blood tissue [46]. Although the number of blood samples in our current database is limited, peripheral samples for the development of biomarkers and individualised therapies may prove to be potent and complementary tools for use in psychiatric research.

Given the importance of the data as a resource for the community focused on psychiatric research, we have made the PD_NGSAtlas publicly available. To build a DNA methylation and gene expression database focusing on human psychiatric diseases, continued efforts will be made to update the PD_NGSAtlas data and improve the genomic viewer and database functionality. In our current study, we also included some sequencing-based DNA methylation and gene expression profiles related to SZ and BP collected from public databases [47]. We will also encourage research scientists to submit their next-generation sequencing data directly to the PD_NGSAtlas and to make this database more comprehensive. The submitted datasets in the future will be manually reviewed and then integrated into this database. In addition, some interfaces are also provided in our current database, and it will be easy to integrate these datasets into the database in the future.

In this study, we proposed the PD_NGSAtlas for the visualisation and analysis of methylation and expression datasets for psychiatric disorders; however, some limitations to the current system need to be addressed in the future. Although a number of datasets were collected and processed into our database, the numbers of samples are still limited. We expected to acquire more samples to make the database more comprehensive in the future. In addition, some statistical methods were incorporated into the database to identify the DEGs. These methods should be used with caution. The user should select the method that is most suitable for a given dataset. For example, the edgeR and DEGSeq methods were specifically incorporated for gene expression profiles based on raw read counts. Moreover, it is notable that the newly submitted datasets were mainly transferred by email to our current database. In response to the rapid increase in the amount of sequencing data produced by the next-generation sequencing technologies, we expect to incorporate more effective methods to enhance the efficiency of this process.

## Conclusions

In this work, we present the PD_NGSAtlas, a specific database for psychiatric disorders, which offers a comprehensive reference resource combining epigenetic and transcriptomic data based on next generation sequencing, and quantitative analysis of epigenetic and transcriptional alterations involved in psychiatric disorders. The PD_NGSAtlas aims to provide reference resources to assist researchers to understand the epigenetic and transcriptional effects involved in the aetiology and pathophysiological mechanisms of psychiatric disorders.

## Availability and requirements

PD_NGSAtlas is freely available at http://bioinfo.hrbmu.edu.cn/pd_ngsatlas/. The web interface has been tested in the following web browsers: Google Chrome (version 17 and later), Apple Safari (version 5 and later) and Mozilla’s Firefox (version 10 and later). The “Help” page of the PD_NGSAtlas Web interface includes a step-by-step description of all PD_NGSAtlas features.

## Additional files

Additional file 1: Figure S1. The overview of the PD_NGSAtlas. (a) The search page of the database shows. (b) The detail page of search gene expression, search methylation peaks and DMRs. (c) The gene expression of specific gene was shown in the search result page. The users can also view the distribution of the gene expression across samples by clicking the bar button. (d) The DNA methylation of specific gene across samples was shown. (e) The identified DMRs across the samples selected by users. (f) The visualization of DNA methylation and gene expression. Additional file 2: Table S1. The clinical characteristics of samples used in PD_NGSAtlas. Additional file 3: Figure S2. Median Phred score vs. base position. The quality scores of the reads were satisfactory, most of the called bases had a Phred score ≥ 30.

## Abbreviations

SZ: Schizophrenia
BP: Bipolar disorder
MBPs: Methyl-CpG binding proteins
BDNF: Brain-derived neurotrophic factor
MZ: Monozygotic
TTUHSC: Texas Tech University Health Science Center
SWBB: Southwest Brain Bank
NOK: Next-of-kin
BA9: Brodmann area 9
BA24: Brodmann area 24
CGIs: CpG islands
