# Supplementary material for: PD_NGSAtlas: a reference database combining next-generation sequencing epigenomic and transcriptomic data for psychiatric disorders
Source: BMC Med Genomics. 2014 Dec 31;7:71. doi: 10.1186/s12920-014-0071-z (PMC4308070; doi:10.1186/s12920-014-0071-z)
Supplement: Additional file 1: Figure S1. — The overview of the PD_NGSAtlas. (a) The search page of the database shows. (b) The detail page of search gene expression, search methylation peaks and DMRs. (c) The gene expression of specific gene was shown in the search result page. The users can also view the distribution of the gene expression across samples by clicking the bar button. (d) The DNA methylation of specific gene across samples was shown. (e) The identified DMRs across the samples selected by users. (f) The visualization of DNA methylation and gene expression. [file 12920_2014_71_MOESM1_ESM.doc]

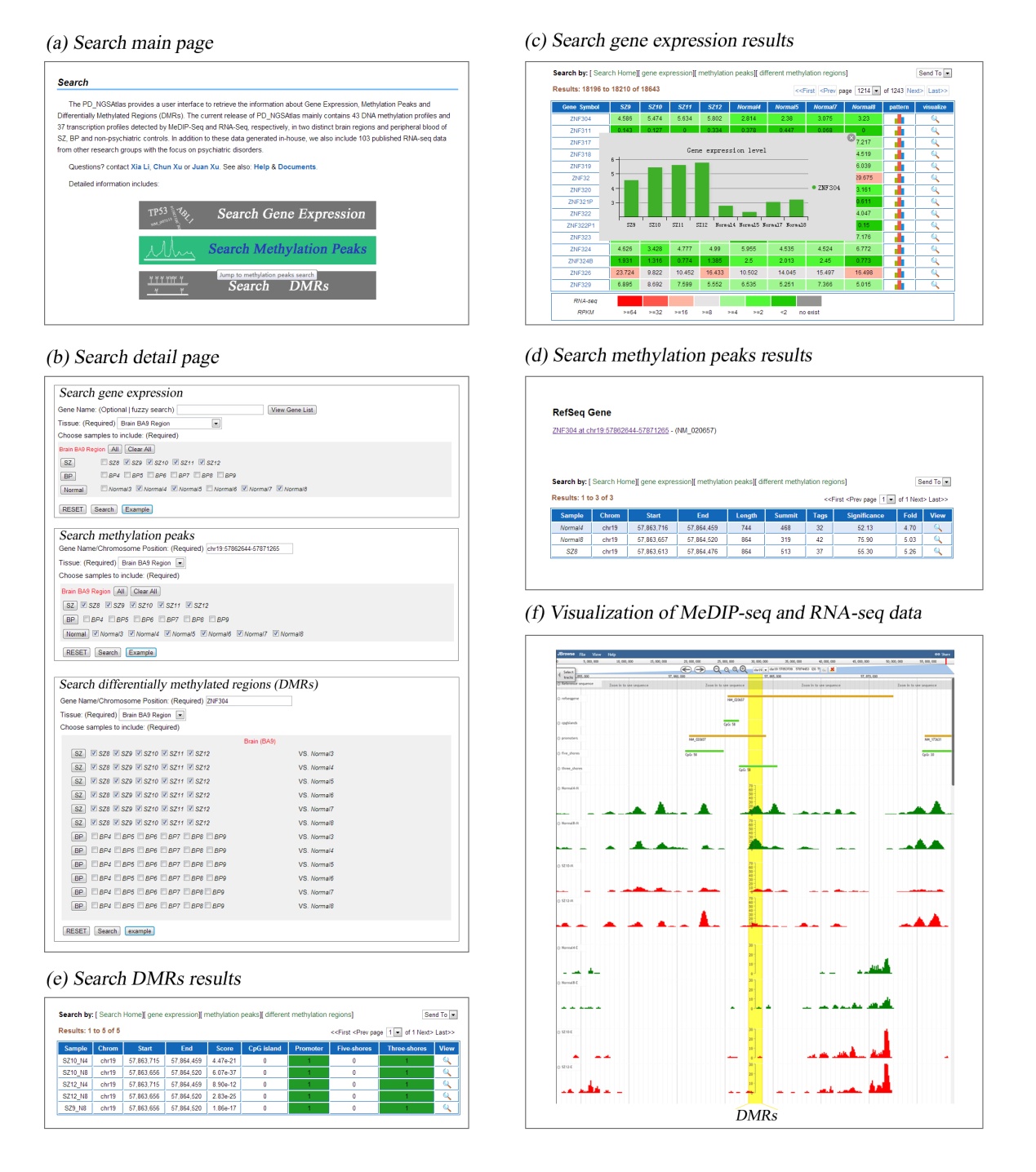


**Figure S1. The overview of the PD_NGSAtlas.** (a) The search page of the database shows. (b) The detail page of search gene expression, search methylation peaks and DMRs. (c) The gene expression of specific gene was shown in the search result page. The users can also view the distribution of the gene expression across samples by clicking the bar button. (d) The DNA methylation of specific gene across samples was shown. (e) The identified DMRs across the samples selected by users. (f) The visualization of DNA methylation and gene expression.
