# Supplementary material for: PD_NGSAtlas: a reference database combining next-generation sequencing epigenomic and transcriptomic data for psychiatric disorders
Source: BMC Med Genomics. 2014 Dec 31;7:71. doi: 10.1186/s12920-014-0071-z (PMC4308070; doi:10.1186/s12920-014-0071-z)
Supplement: Additional file 3: Figure S2. — Median Phred score vs. base position. The quality scores of the reads were satisfactory, most of the called bases had a Phred score ≥ 30. [file 12920_2014_71_MOESM3_ESM.doc]

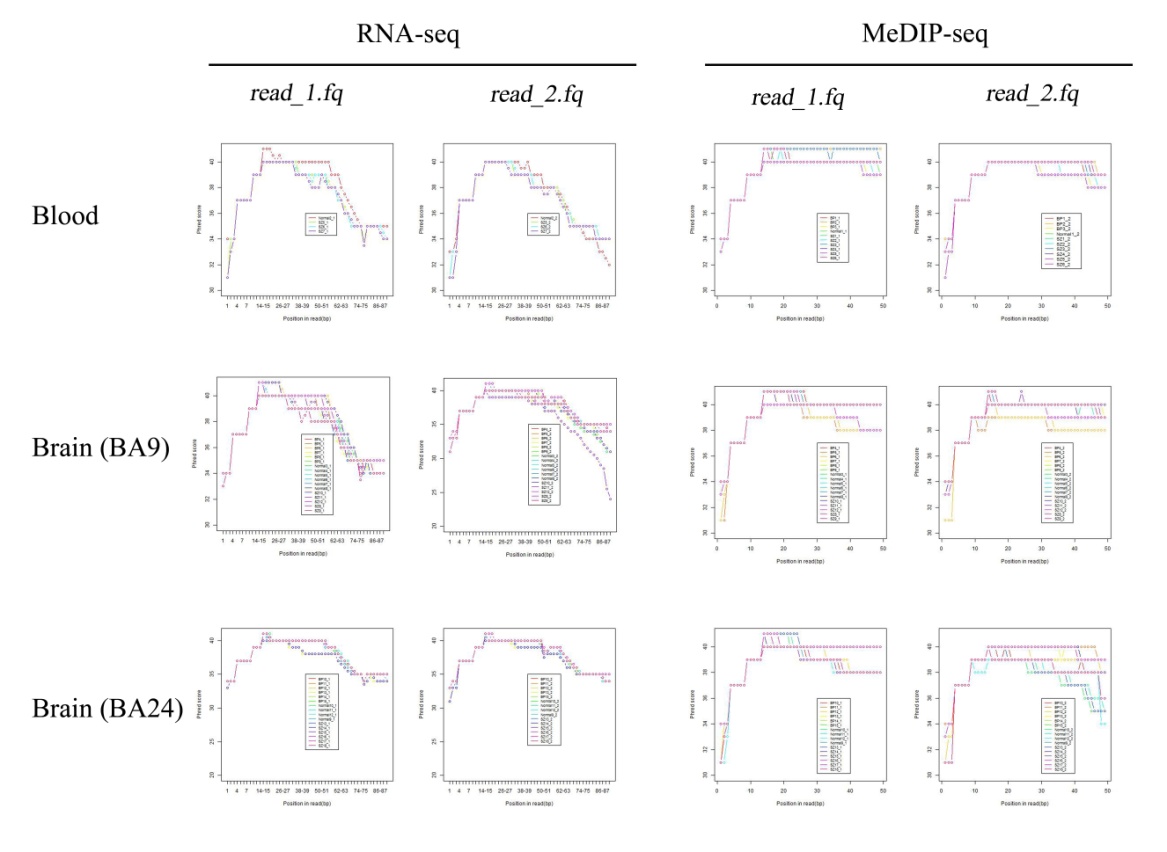


**Figure S2. Median Phred score vs. base position.** The quality scores of the reads were satisfactory, most of the called bases had a Phred score ≥ 30.
